# Epistaxis

**DOI:** 10.1016/S1808-8694(15)30644-3

**Published:** 2015-10-19

**Authors:** João Ferreira de Mello

**Affiliations:** Brazilian Journal of Otorhinolaryngology - Editor Associate Professor – University of São Paulo Medical School

**Keywords:** epistaxis, nasal bleeding, treatment

Winter is right around the corner, a time of the year in which some regions of the country have climatic conditions characterized by low temperatures and low humidity. Moreover, in certain cities there are more pollutants building up in the atmosphere. A consequence of this is that numerous upper airway diseases become frequent. Among them we list: upper airway infections, inflammatory rhinitis and epistaxis.

Nasal hemorrhage is one of the main otorhinolaryngological emergencies. It is estimated that 60% of the population will have epistaxis at some point in their lives; 6% will require medical care to control it; and 1.6 for every 100,000 will be hospitalized.

Nasal hemorrhages are classified as anterior or posterior. The area of Kiesselbach, located in the anterior portion of the nasal septum and anterior portion of the anterior nasal conchae, is the most commonly involved site in anterior epistaxis. On the other hand, posterior bleeding originates from branches of the sphenopalatine artery.

They have many etiologies, such as trauma (by fingernail or facial trauma), dried nasal mucosa, foreign body introduction, inhaling irritating agents, infectious rhinosinusitis, nasal or rhinopharynx tumors (nasoangiofibroma, polyps, neoplasias, etc), vascular alterations (telangiectasia), blood coagulation disorders, etc.

There are three basic goals in its treatment. We must bear in mind the volume of blood lost, pin-point the hemorrhage site and establish the best treatment possible. Most of the times, anterior and/or posterior nasal packing are enough to control the problem. Notwithstanding, in some situations there is the need for more aggressive treatment.

In this issue of our journal we present a study carried out at the Ribeirão Preto Medical School of the University of São Paulo, in which the authors analyzed the causes and evolution of cases of epistaxis resistant to nasal packing.

